# Pimozide as an effective treatment for obsessive symptoms related to physical discomfort in the context of somatoform symptomatology.

**DOI:** 10.1192/j.eurpsy.2024.874

**Published:** 2024-08-27

**Authors:** P. Del Sol Calderon, A. Izquierdo de la Puente, R. Fernández, M. García Moreno

**Affiliations:** ^1^Psychiatry, Hospital Puerta de Hierro; ^2^Psychiatry, Hospital Universitario Infanta Cristina, Madrid, Spain

## Abstract

**Introduction:**

The patient was a 34-year-old male admitted to the psychiatric inpatient unit for high anxiety and suicide ideation due to severe toothache.

**Objectives:**

To show how the antipsychotic pimozide can be an effective option for the treatment of anxiety and obsessive symptoms around physical complaints within the spectrum of somatoform disorders

**Methods:**

Case report and literature review

**Results:**

The patient comes to the emergency room with high anxiety and active self-harming ideation. He reports that for months he has been experiencing mouth pain that is becoming more and more intense. He has seen multiple professionals without finding a cause that justifies the pain. In the past she has a history of multiple ailments (knee, abdominal pain…). He is being treated with sertraline 150, clonazepam 3 mg per day and olanzapine 5 mg at night. During admission, treatment with pimozide up to 4 mg per day was started. The patient is progressively less distressed and with more distance from the ideas about pain, being able to carry out more activities during the day. There is remission of suicidal ideation

**Conclusions:**

There is evidence in the literature that the use of pimozide was effective in different psychotic disorders. It has been seen to reduce the intensity of symptoms in cases of delusional disorders with delirum of somatic type or those such as delusions by parasitization. The use of pimozide has also been effective in the treatment of complex tic disorder. In this case it is effective and could be explained by the close relationship of osbsesive symptoms with psychotic symptoms.

**Disclosure of Interest:**

None Declared

